# Social Anxiety Disorder and Alcohol Use

**Published:** 2002

**Authors:** Sarah W. Book, Carrie L. Randall

**Affiliations:** Sarah W. Book, M.D., is an assistant professor and Carrie L. Randall, Ph.D., is a professor in the Department of Psychiatry at the Center for Drug and Alcohol Programs and Alcohol Research Center, Medical University of South Carolina, Charleston, South Carolina

**Keywords:** comorbidity, anxiety, social phobia, AODU (alcohol and other drug use), alcoholic beverage, tension reduction theory of AODU, diagnostic criteria, prevalence, positive AOD (alcohol and other drug) expectancies, drug therapy, psychotherapy, monoamine oxidase, benzodiazepines, serotonin uptake inhibitors, literature review

## Abstract

Social anxiety disorder—an excessive fear of social situations, such as eating or speaking in public—affects 2 to 13 percent of the U.S. population. About one-fifth of patients with social anxiety disorder also suffer from an alcohol use disorder (AUD) (i.e., alcohol abuse or dependence). One theory to explain the comorbidity between social anxiety disorder and AUDs is the tension reduction theory, which posits that people with social anxiety use alcohol to alleviate their fears. This expectation that alcohol reduces anxiety may motivate alcohol consumption even if pharmacological studies do not support that assumption. Social anxiety disorder is treatable with both pharmacotherapy and psychotherapy, and some of those treatments also would be expected to be effective for patients with comorbid AUDs. Evaluation of patients seeking alcoholism treatment for coexisting social anxiety disorder is important for improving treatment outcome.

Many people experience social anxiety—that is, they feel uncomfortable or even anxious in social situations, such as talking with strangers (or even friends) or speaking in front of a group of people. In the general population, levels of social anxiety exist on a continuum from mild to severe. A clinical diagnosis of social anxiety disorder, also referred to as social phobia,^1^ is assigned only when the social anxiety results in significant fear when faced with the situation, impairment of performance, or avoidance of anxiety-provoking situations. People with high levels of social anxiety typically report that alcohol helps them feel more comfortable in social situations. Thus, it is not surprising that individuals with clinically diagnosed social anxiety disorder have a higher incidence of alcohol-related problems than does the general population. This article explores the diagnosis and prevalence of social anxiety disorder and reviews studies evaluating the relationship between alcohol consumption and social anxiety. In addition, the article summarizes treatment approaches for social anxiety disorder alone as well as in combination with alcohol-related problems.

## Social Anxiety Disorder

According to the American Psychiatric Association’s *Diagnostic and Statistical Manual of Mental Disorders, Fourth Edition* (DSM–IV) (1994), social anxiety disorder is defined as excessive fear in social situations in which the person believes he or she will do something embarrassing or have anxiety symptoms (e.g., blushing or sweating) that will be humiliating. The feared situations can vary from interpersonal social interactions in small groups to talking to strangers. Performance fears, such as speaking in public, also are common. People with social anxiety disorder either avoid feared situations or experience them with extreme anxiety. Most individuals with the disorder have the more severe, “generalized” type, in which the person has other social fears in addition to the common fear of public speaking. The complete criteria set for an official diagnosis of social anxiety disorder is detailed in the textbox.

**Diagnostic Criteria for Social Phobia**

Social phobia* is diagnosed if the person meets all of the following criteria:

- A marked and persistent fear of one or more social or performance situations in which the person is exposed to unfamiliar people or to possible scrutiny by others. The individual fears that he or she will act in a way (or show anxiety symptoms) that will be humiliating or embarrassing.
- Exposure to the feared social situation almost invariably provokes anxiety, which may take the form of a situationally bound or situationally predisposed panic attack.
- The person recognizes that the fear is excessive or unreasonable.
- The feared social or performance situations are avoided or else are endured with intense anxiety or distress.
- The avoidance, anxious anticipation, or distress in the feared social or performance situation(s) interferes significantly with the person’s normal routine, occupational (academic) functioning, or social activities or relationships, or there is marked distress about having the phobia.
- In individuals under the age of 18, the duration is at least 6 months.
- The fear or avoidance is not the result of the direct physiological effects of a substance or a general medical condition and is not better accounted for by another mental disorder.
- If a general medical condition or another mental disorder is present, the fear in Criterion A is unrelated to it.

SOURCE: Adapted from the *Diagnostic and Statistical Manual of Mental Disorders, Fourth Edition* (American Psychiatric Association 1994).

*Social phobia may be specific to one or a few social situations or generalized to most social situations.

Typically, social anxiety disorder begins in the teenage years and does not improve without treatment. The mechanisms or causes underlying the disease are unknown but may involve multiple predisposing factors. These potential factors include genetic background, traumatic early emotional learning experiences, observation and modeling of parental behaviors, and biological irregularities in brain chemical systems. People with the disorder often report having been shy or behaviorally inhibited as small children and, in severe cases, a child with social anxiety may not want to go to school (Beidel 1998).

As young adults, people with social anxiety disorder tend to make life choices based on their fears, such as avoiding classes requiring oral presentations or accepting jobs beneath their ability because they can work alone. Because these life choices circumvent their underlying social fears, many people with social anxiety disorder may be able to function adequately for extended periods of time. Once the contrived situation changes (e.g., when a promotion is offered), however, the social anxiety fears may resurface. Many people with social anxiety disorder will turn down a promotion rather than encounter the social demands required for the new job. Thus, for many of these people, life is orchestrated around social fears, even if this results in social isolation or less financial independence and academic achievement.

### The Prevalence of Social Anxiety Disorder

Prevalence estimates of social anxiety disorder vary considerably, both for social anxiety alone and concurrently with alcohol use disorders (AUDs)^2^ (e.g., Kessler et al. 1997; Schneier et al. 1992; Van Ameringen et al. 1991). This variability results in large part from methodological differences among studies. For example, an analysis of data from the Epidemiologic Catchment Area study, a door-to-door survey conducted in four communities across the United States in the 1970s, found the prevalence of social anxiety disorder to be only 2 percent (Schneier et al. 1992). In this analysis, a diagnosis of social phobia was given to respondents who admitted to extreme anxiety while eating in front of others, speaking to a small group of people they knew, or speaking to a large group of strangers. In contrast, a later community study, the National Comorbidity Survey, found the prevalence of social anxiety disorder to be as high as 13 percent (Kessler et al. 1998). In this study, respondents acknowledging anxiety in a wider range of social situations were considered to have social anxiety disorder. This higher prevalence rate of social anxiety disorder would make it the third most prevalent psychiatric disorder after alcohol dependence and depression.

Conservative estimates of the prevalence of co-occurring, or comorbid, social anxiety disorder and AUDs found that approximately 20 percent of patients treated for social anxiety disorder and 15 percent of people receiving alcoholism treatment have both disorders (Randall et al. 2001*a*). Despite a paucity of controlled research in the area, many researchers have written about the high comorbidity between social anxiety disorder and alcohol use problems. One viable hypothesis to explain this high comorbidity is that people with social anxiety use alcohol as self-medication for social fears. This tension reduction hypothesis is discussed next.

## The Tension Reduction Hypothesis

Clinical reports indicate that people use alcohol as a means of coping with social fears as well as with stress. One standard theory of why people drink— the tension reduction hypothesis— implies that alcohol acts as a negative reinforcer to reduce stress and anxiety. A negative reinforcer is something that eliminates an unpleasant experience. In this case, anxiety or stress is the unpleasant experience and alcohol consumption, which reduces these feelings, would be considered the negative reinforcer. Once a person experiences stress relief after consuming alcohol, he or she is likely to continue to use alcohol for its stress-reducing properties. Whether alcohol actually reduces stress is debatable (see Carrigan and Randall, in press). In fact, some researchers have argued that based on its pharmacological properties, alcohol actually should increase stress and that therefore negative reinforcement using alcohol would be ineffective (Spencer and Hutchison 1999).

Nevertheless, people with social anxiety disorder report that aside from totally avoiding anxiety-inducing situations, alcohol use is one of their primary means of coping. It is possible that positive expectancies—beliefs that alcohol will relieve social anxiety, whether supported in fact or not—play a role in the relationship between social anxiety and alcohol use. For example, people with social anxiety may drink excessively because they strongly expect alcohol to reduce their anxious feelings in social situations. The role of alcohol expectancies and their relation to alcohol consumption and behavior has been studied extensively in young adults (e.g., Burke and Stephens 1999; Tran et al. 1997). These experiments demonstrated that both positive and negative expectations can be powerful behavioral moderators. Differences in expectancies might in fact explain why not all people with social anxiety drink alcohol to cope with their fears. People with negative expectancies about alcohol’s effects probably do not consume alcohol because it increases their fear of appearing even more foolish.

Positive expectancies that alcohol can relieve social fears may explain why some people experiment with alcohol as a coping strategy in the first place. If a person’s expectancy that alcohol reduces stress is left unchallenged (i.e., if the person is not shown by a clinician or researcher that alcohol consumption does not actually reduce stress either behaviorally or physiologically), it may be a powerful enough belief system to explain why a person continues to use alcohol to relieve stress. Alternatively, one can argue that for a subgroup of socially anxious people alcohol may have a genuine pharmacological effect that results in decreased social anxiety. These individuals may have started drinking as a coping mechanism because of their positive expectancies, but they may continue to use alcohol because they associate alcohol consumption with symptom relief. Unfortunately, few experimental studies have investigated expectancies about alcohol’s ability to reduce social fears, determined how expectancies translate into actual alcohol consumption, or demonstrated that alcohol reduces social fears in a laboratory setting (see Carrigan and Randall, in press).

Much of what is known about the association between social anxiety and alcohol consumption comes from research with college students who did not have clinical diagnoses of social anxiety disorder, rather than from research with patients diagnosed as having the disorder. In one study examining the effects of alcohol expectancies on drinking, Tran and colleagues (1997) divided a sample of college students into two groups according to their anxiety level (i.e., high or low) and assessed their expectancies about alcohol’s ability to relieve social anxiety as well as their alcohol consumption. The study found that among those respondents who believed that alcohol would definitely reduce social fears, the level of social anxiety did not affect the level of alcohol consumption. Thus, although the investigators had expected the high-anxiety group to consume more alcohol than the low-anxiety group when alcohol was being used to cope with social fears, no such difference existed, at least in these non-treatment-seeking college students. This lack of a difference might, however, be, in part, the result of the social context of college student drinking. That is, for both groups baseline drinking levels would be expected to be relatively high, to be defined by drinking binges, and to occur in the context of peer pressure and social situations.

Another way to examine the relationship between alcohol expectancies and alcohol consumption is to ask whether people with high expectations of reduced social anxiety actually experience less anxiety when they are drinking. In a sample of 72 female college-age social drinkers, women who expected positive results from alcohol did, in fact, report less anxiety after drinking alcohol than did women with negative or neutral beliefs (de Boer et al. 1994). One can assume that the greater reduction in anxiety after alcohol consumption would result in more drinking in the group with the positive alcohol expectancies. However, the study did not examine drinking behavior, nor did it compare results for women with high or low levels of social anxiety.

Another study assessed drinking in a sample of male college students with high social anxiety and failed to find evidence of increased alcohol consumption compared with students with lower levels of anxiety (Holroyd 1978). In fact, severity of social anxiety was a negative predictor of alcohol quantity consumed— that is, participants with higher levels of anxiety consumed less alcohol than did those with lower levels of anxiety. Unfortunately, however, this study did not examine alcohol expectancies and only sampled males.

Taken together, the results from these last two studies suggest that the interaction of gender and alcohol expectancies may be an important factor in determining who will report that alcohol benefits them and who might drink alcohol to cope with their anxiety.

Whether alcohol reduces social anxiety in a controlled laboratory environment is still open to debate. Several studies have addressed this issue in college students (see Carrigan and Randall, in press), and one study was conducted in a clinical sample of clients with a formal diagnosis of social anxiety disorder (Himle et al. 1999). This latter study did not find that alcohol had any effect in reducing social fears. The conclusions from this study are limited, however, because the investigators excluded patients with a diagnosis of alcohol abuse or dependence and did not evaluate alcohol expectancies. Thus, researchers do not yet know whether alcohol reduces social anxiety in people who believe it is effective or in people who consume it to the point that they meet the criteria for an AUD. The specific alcohol effects observed may also depend on how anxiety is measured (e.g., by behavioral or psychophysiological measures or by self-report).

Regardless of whether researchers can demonstrate in the laboratory that alcohol reduces social fears, many people with social anxiety report that they expect alcohol to have that effect and that they use it to cope with their social anxiety. Because of their alcohol consumption, some of these individuals will eventually develop alcohol use problems in addition to their preexisting social anxiety disorder. For other people, alcohol use as a coping mechanism could still lead to alcohol-related risks, such as automobile crashes, interpersonal conflicts, or medical risks, even if they do not meet the criteria for an AUD. Treatment of social anxiety therefore is warranted to prevent the development of alcohol-related problems and AUDs.

## Treatment of Social Anxiety Disorder

As with most anxiety disorders, social anxiety disorder responds well to pharmacologic treatment and psychotherapy. The aim of both types of interventions is to reduce social fears so that the feared situations are no longer avoided or that they are associated with minimal discomfort. The following sections review various therapeutic interventions.

### Pharmacotherapy

Several different classes of medication have been shown to be effective in the treatment of social anxiety disorder. The primary drug classes include the monoamine oxidase inhibitors (MAOIs), benzodiazepines, and more recently, selective serotonin reuptake inhibitors (SSRIs) (Blanco et al. 2002).

#### MAOIs

These medications block monoamine oxidase, an enzyme that breaks down monoamines. Monoamines are one of the major classes of signal-transmitting chemicals (i.e., neurotransmitters) in the brain and are thought to play a role in mood and anxiety disorders. MAOIs have been shown to be efficacious in the treatment of anxiety disorders in several well-designed studies (Blanco et al. 2002). However, these medications require a special diet that restricts the ingestion of the amino acid tyramine (which is contained in cheese, red wine, some imported beers, and yogurt) to avoid a potentially deadly episode of high blood pressure (i.e., a hypertensive crisis). Because of this potentially serious side effect, most physicians no longer prescribe these medications.

#### Benzodiazepines

These sedative medications are also effective in the treatment of social anxiety disorder. For example, alprazolam and clonazepam are both well tolerated and have shown promise in the treatment of social anxiety (Blanco et al. 2002). However, as discussed more fully below, their use in patients with comorbid social anxiety and alcohol problems may not be advisable.

#### SSRIs

These agents, which affect a brain signaling system using the brain chemical serotonin, are perhaps the best tolerated and most carefully studied pharmacologic treatment for social anxiety disorder. Although several of these medications have shown some efficacy, paroxetine (Paxil^®^) is the only medication currently on the market that has been approved by the U.S. Food and Drug Administration for the treatment of social anxiety disorder. At least three clinical trials have shown this agent to be significantly more effective than an inactive substance (i.e., a placebo) in reducing social anxiety and alleviating the problems associated with social anxiety disorder (Baldwin 2000). Based on these results, SSRIs have been proposed as the first line of treatment for social anxiety disorder (Blanco et al. 2002).

### Psychotherapy

Psychological treatment of people with social anxiety disorder has used various approaches that focus on role playing, coping skills training, exposure to feared situations, and changing the patients’ erroneous negative beliefs about how people perceive them (Heimberg 2002). Because social anxiety disorder involves excessive fear of humiliation and criticism, cognitive behavioral therapies (CBT) have proved to be successful for this disorder and, like medications, to be more effective than control conditions (Heimberg et al. 1998). CBT approaches are based on the assumption that human behavior is largely learned and that learning processes therefore can be used to change problem behaviors. CBT is not one uniform type of therapy but can be classified into four different categories (Heimberg 2001):

- *Exposure therapy* is a technique in which patients are given skills to alleviate anxiety and then are encouraged to expose themselves to an anxiety-provoking situation in an attempt to extinguish the anxiety. Exposure to anxiety-inducing situations is considered an essential component of therapy because many patients, if left on their own, would avoid such situations altogether, thereby restricting their lifestyle.
- *Cognitive restructuring* is a model in which clients are taught to identify thoughts that provoke anxiety and to think in a less biased, anxiety-provoking way. Challenging erroneous beliefs with video feedback or feedback from group members is an important component of cognitive therapy.
- *Relaxation training* is a method of decreasing anxiety by controlling the amount of physical tension experienced in a stressful situation. Relaxation training alone, however, is not usually as successful as cognitive restructuring or exposure therapy.
- *Social skills training* teaches patients how to improve their behavioral skills in social interactions. However, not all people with social anxiety disorder have deficits in social skills. Therefore, social skills training alone would not benefit all clients with the disorder.

Most CBT for social anxiety disorder involves a combination of these different approaches. Regardless of which specific CBT measures are evaluated, it is important to recognize that non-pharmacological treatments for social anxiety disorder are effective (Taylor 1996) and generally work as well as medication. In addition, some findings indicate that despite a slower onset of improvement compared with pharmacotherapy, the beneficial effect of psychotherapy may be more enduring (Heimberg 2002).

## Treatment of Comorbid Social Anxiety Disorder and AUDS

The previous discussion indicates that social anxiety disorder is treatable, at least in people without comorbid alcohol problems. Unfortunately, all of the above-mentioned clinical trials evaluating social anxiety therapies have excluded participants who met the diagnostic criteria for an AUD. Consequently, it is unknown if similar approaches are efficacious in clients with comorbid AUDs and social anxiety disorder.

### Pharmacotherapy

Some treatments are contraindicated for people with AUDs for safety reasons. For example, as mentioned previously, patients taking MAOIs must avoid foods containing the amino acid tyramine, which include certain alcoholic beverages. Therefore, MAOIs could be particularly dangerous for patients with AUDs. Benzodiazepines also should not be used in clients with AUDs for several reasons. First, benzodiazepines can cause behavioral disinhibition, which may make it more difficult for a person to limit their alcohol consumption to an intended amount. Second, benzodiazepines and alcohol affect some of the same brain areas, including a region responsible for maintaining breathing activity. Therefore, people who mix alcohol and benzodiazepines may inadvertently shut down that brain area, with potentially deadly consequences. Finally, people who abuse or are dependent on alcohol are also at risk for abusing or becoming dependent on benzodiazepines.

Only a few studies have evaluated treatment of people with comorbid social anxiety disorder and AUDs, but based on research in individuals with social anxiety disorder alone, some treatments would be expected to be efficacious. Of the pharmacological treatments, SSRIs may be most appropriate for clients with both types of disorders. These medications are safe, are not addictive, and do not interact with alcohol in the brain. In addition, accumulating evidence suggests that SSRIs also have some efficacy in improving outcome in treatment-seeking alcoholics with a late onset of alcoholism (i.e., after age 25) (Pettinati et al. 2000), which includes most socially anxious alcoholics.

Randall and colleagues (2001*b*) recently completed the only study to date that examines the use of SSRIs by clients with comorbid social anxiety disorder and alcoholism. The study was an 8-week, placebo-controlled pilot clinical trial comparing the efficacy of paroxetine to placebo. Results indicated that paroxetine not only reduced social anxiety to a significant degree but also tended to decrease the frequency and severity of alcohol use. A larger trial to replicate and extend these findings is currently under way.

### Psychotherapy

Whether CBT is beneficial in patients with comorbid social anxiety disorder and AUDs is not clear.^3^ A recent randomized, controlled clinical trial failed to demonstrate any benefit when the patients received simultaneous CBT for each condition (Randall et al. 2001*a*). In fact, treatment outcome was slightly better in the group that only received CBT for their alcohol problems than in the group that received CBT treatment for both conditions. These results highlight several important factors that must be addressed when treating individuals suffering from more than one psychiatric disorder. For example, which condition should be treated first? Should the treatments be integrated or separated? Should different treatment approaches be used (e.g., pharmacotherapy for one disorder and psychotherapy for another)?

It is likely that the answers to these questions depend at least in part on the problem for which a given patient seeks treatment. For example, for teenagers and young adults, who generally use alcohol only as a coping strategy, treatment primarily of the social anxiety disorder is desirable to prevent the development of further AUDs. Once an AUD has developed, treatment of the social anxiety is likely to be more difficult because each disorder will have a life of its own.

Treatment-seeking alcoholics are rarely assessed for the presence of social anxiety. Alcoholics with comorbid social anxiety disorder, however, are unlikely to benefit as much from traditional alcoholism treatment if their anxiety disorder is not treated. For example, such clients will probably resist or avoid participation in group therapy or self-help meetings, such as Alcoholics Anonymous. This resistance may be especially common in women with social anxiety (Thevos et al. 2000). Thus, in order to improve treatment outcomes it is important to determine whether comorbid social anxiety disorder is present among people seeking alcoholism treatment (Randall et al. 2000). It is equally important to inquire about alcohol consumption in people seeking treatment for social anxiety problems in order to improve the outcome of treatment for social anxiety as well as to prevent or identify alcohol-related problems.

## Summary

The discussion presented in this article clearly indicates that the data associating drinking with relief of social anxiety are complex and inconclusive. Studies in college students with a certain level of social anxiety did not support the assumption that drinking alcohol can indeed relieve social anxiety; however, these students did not have a formal diagnosis of social anxiety disorder. The only study involving individuals meeting the diagnostic criteria for social anxiety disorder excluded clients who met the criteria for an AUD—a group who possibly would be expected to experience relief of social anxiety symptoms after alcohol consumption.

Regardless of whether some people experience actual benefits from alcohol consumption, the belief that alcohol may reduce social anxiety motivates many socially anxious people to use alcohol as a primary coping strategy. This is especially true for individuals who are still engaging in social interactions and are not yet avoiding them completely.

As described above, social anxiety disorder is treatable. If it is left untreated, however, repeated use of alcohol to self-medicate may result in the development of a secondary AUD, so that the individual suffers from two comorbid disorders requiring treatment. The onset of social anxiety disorder nearly always precedes the onset of an AUD, often by more than 10 years (Randall et al. 2001*a*). Because of this delay, this particular comorbidity is a primary target for intervention in order to prevent the development of an AUD.

## Footnotes

1 These two terms tend to be used interchangeably in the literature.

2 Alcohol use disorders as defined in the DSM–IV include alcohol abuse and alcohol dependence.

3 CBT also is frequently used in the treatment of AUDs, although the specific treatment approaches used and their target behaviors differ from those used for clients with social anxiety disorder.
